# Supplementary material for: Sleep drive, not total sleep amount, increases seizure risk
Source: Nat Commun. 2025 Jul 29;16:6967. doi: 10.1038/s41467-025-62311-x (PMC12307685; doi:10.1038/s41467-025-62311-x)
Supplement: Supplementary file 1 — Supplementary Information [file 41467_2025_62311_MOESM1_ESM.pdf]

Supplementary Methods

Drosophila melanogaster

Several existing mutant fly lines were used including two bang-sensitive mutants (*tko*^{25t} and *eas*^{pc80f}), two short-sleeping mutants (*rye* and *sss*^{p1}), and *nemuri* mutants (*nur*^{3}). Wild-type Canton-S flies were used as unless otherwise indicated. UAS, Gal4, lexA, and lexAOp fly lines already not in lab or gifted were obtained from Bloomington Drosophila Stock Center (BDSC) in Indiana, USA or Vienna Drosophila Resource Center (VDRC) in Austria. See below for details on where each line was obtained from.

The following fly stocks were used in this study: *iso*^{31} (control strain, lab stock), Canton-S (control strain, lab stock), *redeye* [1] (lab stock), *tko*^{25t} [2] (gift from Dr. Dan Kuebler), *para*^{bss1} (gift from Dr. Dan Kuebler), *eas*^{pc80f} (lab stock), *sleepless* P1 [3] (lab stock), 60D04-Gal4 (BDSC #45356), nsyb-Gal4, (lab stock), 11H05-Gal4 (BDSC #45016), UAS-TrpA1 (II) [4] (gift from Dr. Leslie Griffith), R23E10-Gal4 (BDSC #49032), UAS-nGFP (BDSC #4775), w; UAS-CD8::RFP, LexAop-CD8::GFP-2A-CD8::GFP; UAS-mLexA-VP16-NFAT, LexAop-CD2::GFP [5, 6] (UAS-CaLexA; lab stock), c453-Gal4 (lab stock), c584-Gal4 (lab stock), Tdc2-Gal4 (lab stock), C929-Gal4 (lab stock), Ddc-Gal4 (lab stock), TH-Gal4 (BDSC #8848), trh-Gal4 (BDSC #38389), 104y-Gal4 (lab stock), 104906-Gal4 (lab stock), 201y-Gal4 (lab stock), kurs58-Gal4 (BDSC # 80985), R58H05-Gal4 (BDSC # 39198), UAS-GCaMP7b (BDSC # 80907), 23E10-lexA (lab stock), LexAOp-csChrimson (lab stock), UAS-csChrimson (lab stock), UAS-GtACR1 (BDSC # 92983), UAS-5HT1A RNAi (VDRC #106094), *nur*^{3} [7] (lab stock), UAS-nemuri [7] (lab stock), elav-GeneSwitch, (lab stock).

Induced seizure analysis

For induced seizures, bang-sensitive mutant flies were placed in groups of 4 flies into vials containing agar (2%) (Fisher NC1429200) and sucrose (5%) 24-hours prior to seizure induction. This was done because anesthetization of flies with CO_2 shortly before seizure induction caused flies to be refractory to seizures. Vials of all experimental conditions were struck on a countertop together four times. All vials were then vortexed on a Fisher Vortex Genie 2 (#12-812) at setting '5'. *tko*^{25t} flies were vortexed for 5 seconds. *eas*^{pc80f} flies were vortexed for 5 seconds. Vials were then placed in front of a USB camera with varifocal manual lens (Mokose #UC70-6-12MM) with video recording on a PC using OBS Studio 29.0. Videos were then subsequently analyzed for percentage flies with seizure in each vial, duration in atonic ("paralysis") phase, duration in tonic/clonic ("convulsive") phase, and duration in postictal ("recovery") phase for each individual fly. Scorers were blinded when possible.

For drug treatment experiments, prior to seizure induction, flies were treated with vehicle, caffeine (1 mg/ml for *tko*^{25t} flies and 0.5 mg/ml for *eas*^{pc80f} flies) [8], or gaboxadol (0.1 mg/ml) for 48 hours with drug mixed in agar (2%) and sucrose (5%). For *tko*^{25t}; elav-GeneSwitch>UAS-nemuri experiments, experimental flies with genetic controls were treated with RU486 (500 µM) for 72 hours prior to experiments.

For the starvation assay, control flies were placed on agar (2%) and sucrose (5%) while starved flies were placed on agar (2%) only for 12 hours as previously reported [9] from ZT12-24. Seizures were subsequently induced between ZT0-1 the following day, and seizure likelihood and duration were measured.

For thermogenetic sleep deprivation, *tko*^{25t}; c584-Gal4>UAS-TrpA1, *tko*^{25t}; Tdc2-Gal4>UAS-TrpA1, *tko*^{25t}; c453-Gal4>UAS-TrpA1, or *tko*^{25t}; 104906-Gal4>UAS-TrpA1 flies with genetic controls were raised at 18°C, maintained at 30°C for 24 hours, then allowed to equilibrate for 20 minutes at room temperature prior to mechano-sensitive seizure induction.

For TrpA1 [4] screening of sleep or wake promoting cells and circuits that affect seizure severity, experimental flies with genetic controls were raised at 18°C. Flies were then brought to 25°C for 4 minutes by immersing vials into a water bath. Vials were then quickly removed from the water bath for mechanical seizure induction, then returned to the water bath at 25°C with video recording of seizure percentages and durations.

Sleep quantification using multibeam infrared monitors

Flies were placed into locomotor tubes containing agar (2%) and sucrose (5%) with vehicle, caffeine (1 mg/ml for *tko*^{25t} flies and 0.5 mg/ml *eas*^{pc80f} flies), or gaboxadol (0.1 mg/ml).

Sleep was measured by counting the number of infrared beam breaks in *Drosophila* activity monitors, specifically the DAM5H multibeam monitor (Trikinetics). Locomotor data were collected using DAMsystem software (Trikinetics). Both 'Movement' and 'Counts' were assessed and extracted from raw files using DAMfilescan (Trikinetics). Sleep was defined as five minutes of inactivity [10, 11]. Sleep was then quantified using Insomniac 3.0 software [12].

For *tko*^{25t}; elav-GeneSwitch>UAS-nemuri experiments, experimental flies with genetic controls were treated with RU486 (500 µM) for 72 hours prior to sleep assessment. Locomotor tubes containing agar (2%) and sucrose (5%) with vehicle or RU486 (500 µM) were used.

Video tracking analyses of spontaneous seizures with CynthiSeize algorithm and sleep

Flies were placed into 24- or 48-well plates containing agar (2%) and sucrose (5%) with vehicle or drugs mixed into the agar. Caffeine was used at 1 mg/ml [8]. Picrotoxin dosing was determined with a toxicity assay; for assays without caffeine, 0.5 mg/ml of picrotoxin was used, and for assays with caffeine, 0.05 mg/ml was used. Levetiracetam was used at 5 mg/ml as previously reported [13]. Sodium valproate was used at 0.5 mM as previously reported [14, 15]. 8-OH-DPAT, a selective 5HT1A receptor agonist, was used at 3 mM [16, 17]. Buspirone dosing was determined with a toxicity assay and used at 3 mM (Supplementary Fig. 17f). Plates were then sealed with a plastic film (PerkinElmer TopSeal A Plus; 6050185), and small holes were placed through the film using a syringe to allow for air and humidity exchange. Plates were then placed in an incubator with light, temperature, and relative humidity control and illuminated with an infrared (IR) light. An IR camera (monochrome GigE camera with IR pass filter) was used for continuous monitoring through alternating 12-hour light:dark cycles at 25 frames per second for 96 hours per experiment.

EthoVision XT (Noldus Information Technology) was used to convert fly positions to XY coordinates over time. Behaviorally, seizures in flies manifest in stereotyped, repetitive tonic-clonic movements that are clearly discernable from typical fly behavior. We operationalized a tonic-clonic movement as a "hyperkinetic event" (HE). Using the "multi-condition" function in EthoVision XT, we defined HE as movements that met the following parameters: velocity = >1.5 cm/sec, acceleration = > 25 cm/sec^2, mobility = >90%, mobility state = highly mobile >80%, and movement = 1.5 cm/sec, 1.0 cm/sec. These parameters were defined through manual review of videos allowing for identification of spontaneous seizures. These HE over time, as well as XY position data, were exported from EthoVision XT and imported into MatLab to implement the

“CynthiSeize algorithm”. Through further manual review of seizure videos, we found that seizures typically manifest as a high density of repeated HE: at least 5 HE occurring in 50 seconds. CynthiSeize used a rolling time window to identify at least 5 HE occurring in 50 seconds. We found through iterative manual review that depending on the etiology of epilepsy (e.g. bang-sensitive mutant fly versus picrotoxin) and corresponding genetic background, the minimum number of HE defining a seizure was variable. . For bang-sensitive flies (tko^{25t} and eas^{pc80f}), spontaneous seizures exhibited at least 7 HE per seizure. For picrotoxin-treated flies, seizures exhibited at least 10 HE per seizure. In all cases, seizures that occurred within 15 minutes of each other were regarded as the same seizure. Wild-type flies (Canton-S, w^{1118}, and iso^{31}) on vehicle (no caffeine or picrotoxin) never exhibited movements that were defined as seizures.

Movement was quantified as previously described [18]. Sleep was defined as inactivity for 5 minutes or more. Sleep or wake status was determined at the time of seizure occurrence and output through the CynthiSeize algorithm. Time since state change is defined as time since a change from sleep-to-wake status or wake-to-sleep status. Sleep history is reported as the percentage of time asleep in the preceding 180 minutes. At https://zenodo.org/records/15619740 we provide step-by-step instructions with screenshots, as well as a sample dataset and sample outputs.

For experiments involving optogenetic stimulation, experimental flies with genetic controls were raised in the dark. (1) For csChrimson experiments, flies were then placed in all-trans retinal (ATR) (300 µM) for 48 hours with 12-hour light:12-hour dark entrainment in blue light. After entrainment, flies were placed into 24- or 48-well plates containing picrotoxin, ATR, and caffeine with stimulation with red light for the first five minutes of every hour around the clock. (2) For GtACR1 experiments [19], flies were placed in all-trans retinal (ATR) (1 mM) for 48 hours with 12-hour light:12-hour dark entrainment in red light. After entrainment, flies were placed into 24- or 48-well plates containing picrotoxin, ATR, and caffeine with stimulation with green light for the first five minutes of every hour around the clock.

<u>TRIC-luciferase assay</u>

The nsyb-Gal4 driver was used to express UAS-TRIC-luciferase pan-neuronally. Adult flies aged 5-10 days were raised in a 12-hour light:12-hour dark cycle. Flies were fed 2 mM luciferin for 24 hours prior to the start of the experiment. Flies were then placed into a 96-well plate containing 100 uL of the following: agar (2%), sucrose (5%), luciferin (2 mM; Gold BioTechnology, Inc.), and picrotoxin (0.5 mg/mL). A clear adhesive plastic film (Top-Seal-A; Perkin Elmer) was used to cover the 96-well plate. Plates were then loaded into a Cytation 5 multimode reader (BioTek) with imaging of luminescence occurring through the thin agar layer and imaging of fly posture through the clear adhesive plastic film. Luminescence was detected with PMTs every 14 minutes. Bright field images were acquired with a 4x objective.

<u>CaLexA imaging</u>

For drug treatment, flies were treated with vehicle or caffeine (1 mg/ml) mixed into agar (2%) and sucrose (5%) [8] for 48 hours. For mechanical sleep restriction, flies were shaken on a pre-programmed vortexer for 12 hours overnight. Adult fly brains were dissected in cold phosphate buffered saline (PBS) and then fixed in 2% paraformaldehyde for 40 minutes at room temperature. Brains were then washed twice for 20 minutes in PBS with 0.3% Triton-X (PBST). Samples were then placed in 50% glycerol and mounted in Vectashield: H1000. Both control and experimental brain were mounted on the same slide for visualization. Primary GFP and RFP signal was

visualized without signal amplification with antibodies. On the same day as mounting, brains were visualized on a Leica Stellaris 8 confocal microscope. Identical settings were used for laser intensity and gain for control and experimental conditions. After image acquisition, Fiji was used for image processing and analysis. All data are presented as GFP:RFP ratios. Whole brain calcium levels are presented as total signal from z-projections. Subregion analyses were performed as measurements of ROIs in individual slices.

RNA sequencing of the dorsal fan shaped body

To isolate and sort dFB neurons, we first generated flies with nuclear GFP expression using the R23E10-Gal4 (BDSC #49032) driver crossed with UAS-nGFP (BDSC#4775). Male flies aged 5-7 days were subjected to sleep deprivation overnight or regular nighttime sleep in the same incubator, and brains were dissected the next day at ZT0. The brains were dissociated using a protocol from Hongjie Li et al. 2018; briefly, flies were dissected in Schneider's medium, followed by dissociation in Papain solution and filtration through a 100 μm cell strainer. The ventral nerve cords were not included. The resulting cells were then suspended in Schneider's medium, and 100 GFP+ cells from each condition were sorted using either BD FACSMelody or BD FACSAria (BD Biosciences). Dead cells were excluded using 4', 6-diamidino-2-phenylindole (DAPI). Doublets were also excluded based on forward scatter (FSC-H by FSC-W) and side scatters (SSC-H by SSC-W). FSC-A determined the size of cells, and validation was done using cells from flies expressing nSyb>nGFP. The sorted cells were frozen and placed into a 96-well plate with lysis buffer from the Smart-seq2 HT kit.

The sorted cells were sent to Admera Health (admerahealth.com) for RNA extraction, RNA library construction, and sequencing using the Smart-seq2 HT kit. The sequencing data were then mapped to the fly genome (BDSG6) using Hisat2 (daehwankimlab.github.io/hisat2), and the alignment results were counted by LiBiNorm tool (warwick.ac.uk/fac/sci/lifesci/research/libinorm) based on the reference genome from GENCODE. Both raw count and TPM (transcripts per million) data were used separately in further analysis. The raw count data were analyzed by IDEP v0.95 (bioinformatics.sdstate.edu/idep) for genes expressed differentially. Genes with CPM > 0.5 were detected in at least three independent samples, and missing values treated as gene median was selected to filter out low-expressed genes. The regularized log transformation was applied to remove the dependence of the variance on the mean. The transformed raw count data were then used for further clustering and PCA. Differentially expressed genes were identified using DESeq2 with an FDR cutoff of 0.1 and minimum fold change of 2.

Quantification and statistical analysis

Pre-testing with Shapiro-Wilk test and Kolmogorov-Smirnov tests were conducted to assess normality and the choice of parametric or non-parametric testing. For two groups, unpaired or paired two-tailed t-test was used for data that were reasonably assumed to be approximately normally distributed. For two groups, if the variance was significantly different, unpaired two-tailed t-test with Welch's correction was used. When comparing two groups, a Mann-Whitney test was used if the normality assumption was not justified, including for ordinal data. When comparing a single control group to multiple experimental groups, a one-way ANOVA with Dunnett's multiple comparisons test was used for approximately normally distributed data, and a Kruskal-Wallis with Dunn's multiple comparisons test was used if the normality assumption was not justified, including for ordinal data. When comparing three or more groups with multiple

comparisons, a one-way ANOVA with Tukey's multiple comparisons test was used for normally distributed data, and a Kruskal-Wallis with Dunn's multiple comparisons test was used when the normality assumption was not justified. If the variance was significantly different between groups when comparing three or more groups using Bartlett's test, one-way ANOVA with Dunnett's T3 multiple comparisons test was used with individual variances computed for each comparison. The number of flies awake or asleep when seizures occur is reported as a percentage with 'pWake' representing the number of flies awake at seizure onset/total number of seizures, and 'pSleep' representing the number of flies asleep at seizure onset/total number of seizures.

For hypothesis testing of spontaneous seizure frequency, given the categorical variable (seizure count per day per fly), right-skewed distribution of this dataset, and censoring due to fly death during the experiment, a negative binomial model with Wald test was implemented. This analysis accounted for lethality observed after picrotoxin-induced seizures. Exploratory analyses implementing negative binomial models were run (1) to assess the effects of 8-OH-DPAT on seizure frequency for each genotype (Fig. 7f) and (2) to assess the effects of buspirone on seizure frequency with and without caffeine (Fig. 8e). For hypothesis testing of seizure duration and hyperkinetic events per seizure, in datasets where one fly experienced multiple seizures, a mixed-effects model was implemented to account for both intra- and inter-fly variability. To reduce the skewness of the original data, p-values were calculated using log-transformed seizure duration and hyperkinetic event count.

Mean and standard error of the mean are used to visually represent the data. Additional details about the sample size (n) for each experiment, statistical testing, and p-values are provided in the Figure Legends. GraphPad Prism was used for all statistical analyses, except for significance testing of spontaneous seizure frequency, seizure duration, and hyperkinetic events per seizure, which were performed in R version 4.2.1.

Supplementary Discussion

In *Drosophila*, across the 3 neurogenetic and 1 pharmacological models tested, most seizures occurred during wakefulness (Supplementary Fig. 7b, f, Supplementary Fig. 8d, Supplementary Fig. 15a, Supplementary Fig 17a, e). We find that the wake state correlates with a history of increased wakefulness (Supplementary Fig. 8d, e), so whether the wake state or a history of wakefulness drives seizures during wakefulness in *Drosophila* remains unclear. The occurrence of seizures during wakefulness or sleep is also likely tied to etiology, e.g., nocturnal seizures are associated with sleep-related hypermotor epilepsy due to pathogenic variants in *CHRNA4* [20, 21]. Our findings also indicate that seizures occurring after recent transitions from wake → sleep or sleep → wake are more likely to be non-lethal (Supplementary Fig. 8f). This is consistent with the finding that flies that have been awake for longer periods of time are more likely to have severe lethal seizures (Supplementary Fig. 8b).

If sleep loss leading to sleepiness worsens seizures, then this raises the interesting possibility that enhancing sleep to dissipate sleepiness might be protective against seizures. Therefore, we attempted two manipulations to increase total sleep: gaboxadol feeding (Fig. 1) and *nemuri* overexpression (Supplementary Fig. 3). While both manipulations increased sleep, there were seemingly opposite effects on seizures; gaboxadol worsened seizures (Fig. 1i) while *nemuri* overexpression was strongly protective (Supplementary Fig. 3d-i). We propose this can be explained by variable effects on sleepiness. The sleep induced by gaboxadol is known to be different from spontaneous sleep as measured by local field potentials in *Drosophila* [22] and

murine electroencephalography [23]. We suggest that the sleep induced by gaboxadol is not the same as spontaneous restorative sleep and is not able to fully dissipate sleep pressure. Therefore, ongoing sleep pressure led to the worsened seizures we observed after gaboxadol treatment. In contrast, *nemuri* overexpression is known to increase both sleep amount and promote deeper sleep depth [7]. In this deep sleep state induced by *nemuri* overexpression, sleep drive may not accumulate suggesting a protective effect against seizures.

Supplementary Figures

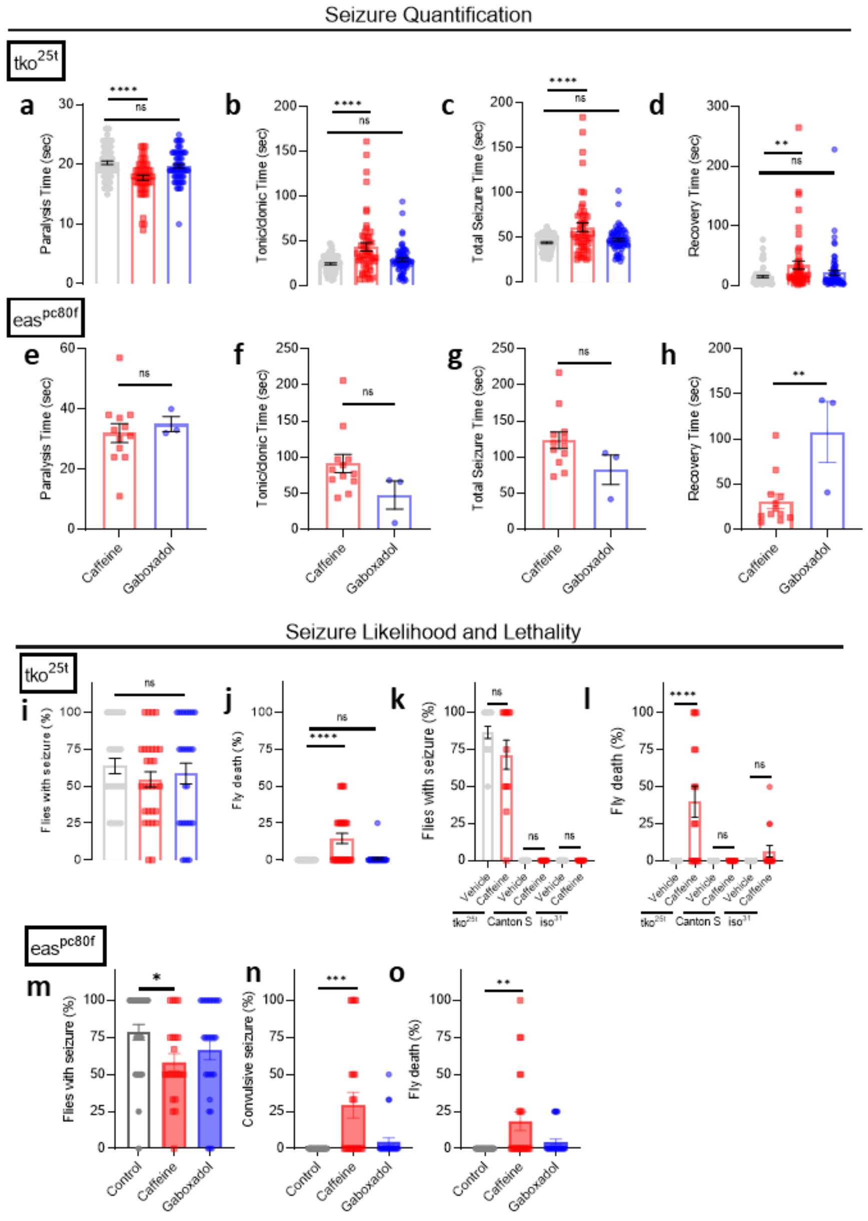

Supplementary Fig. 1 │ Caffeine leads to more severe induced seizures and death in bang-sensitive mutant flies. a-d, Compared to control, *tko*^{25t} flies exhibit decreased mean paralysis time and prolonged tonic/clonic, total seizure, and recovery time with caffeine treatment (*red*). Compared to control, gaboxadol treatment does not significantly change mean seizure times. n = 51-74 flies/condition. **e-h**, In *eas*^{pc80f} flies, there is no significant evidence of differences in mean paralysis time, tonic/clonic time, and between caffeine and gaboxadol treatment. Recovery times are significantly prolonged with gaboxadol treatment as compared to caffeine treatment. Vehicle-fed *eas*^{pc80f} flies did not exhibit tonic/clonic seizures, therefore individual "paralysis time", "tonic/clonic time", "total seizure time", and "recovery time" could not be calculated. n = 43-88 flies/condition. Note there are far fewer tonic/clonic seizures among *eas*^{pc80f} flies, and thus the

statistical power to detect differences is lower than for a-d. **i-j**, We did not observe differences in induced seizure likelihood after caffeine treatment for 48 hours among *tko*^{25t} flies, but flies were more likely to die. n = 29 vials/condition. **k-l**, *tko*^{25t} flies had comparable numbers of seizures but were more likely to die after caffeine treatment for 60 hours. Canton S and iso^{31} wildtype flies never exhibited seizures and are less likely to die after caffeine treatment for 60 hours. n = 12-15 vials/condition. **m-o**, *eas*^{pc80f} flies are less likely to exhibit induced seizures and more likely to die after caffeine treatment for 48 hours. When seizures do occur, *eas*^{pc80f} flies are more likely to exhibit convulsive (tonic/clonic) seizures after caffeine treatment. n = 21-28 vials/condition. Two-group two-tailed t-test, unpaired two-tailed t-test with Welch's correction, one-way ANOVA with Dunnett's multiple comparisons test, or Kruskal-Wallis with Dunn's multiple comparisons test (for seizure percentage or fly death percentage) was used. *p<0.05, **p<0.01, ***p<0.001, ****p<0.0001. Data are presented as mean values ± SEM.

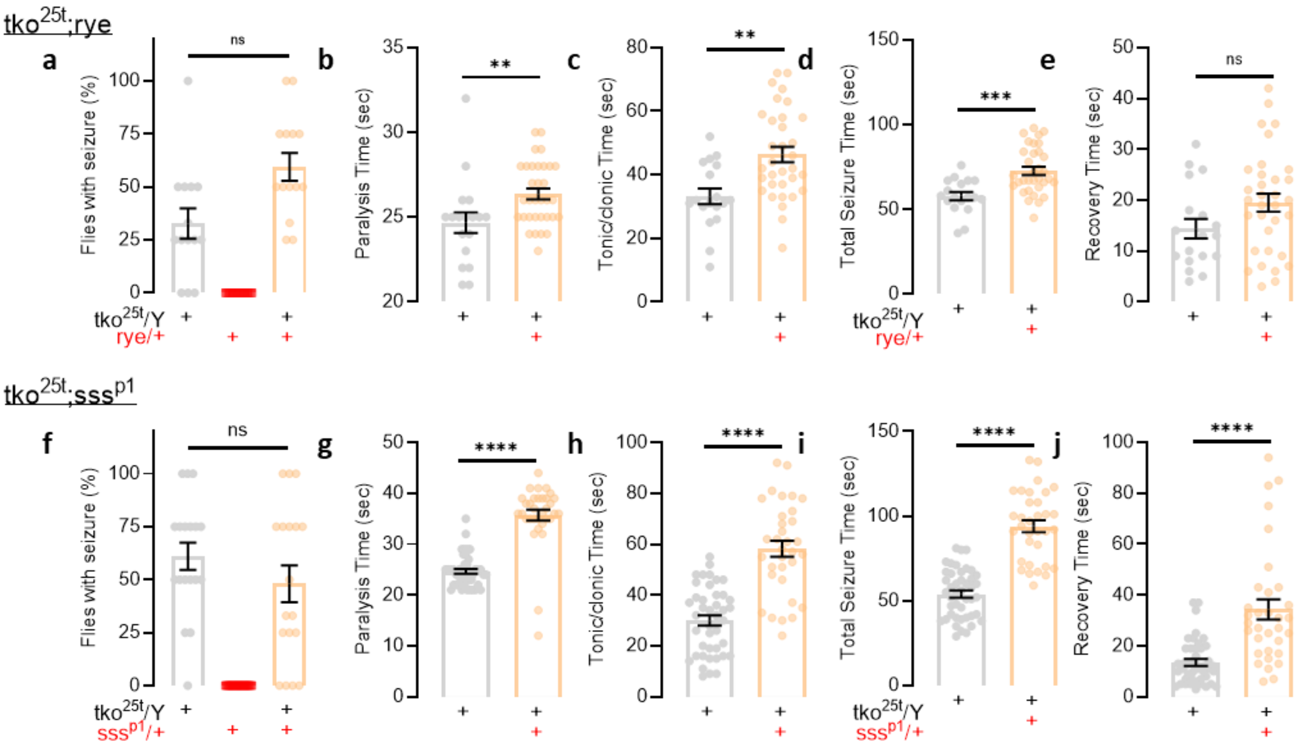

Supplementary Fig. 2 │Crossing short-sleeping flies with *tko^{25t}* mutant flies leads to more severe induced seizures. **a-e**, *tko^{25t};rye* flies have more severe seizures as compared to mutant *tko^{25t}* flies. n = 14 vials/condition and n = 18-33 flies/condition. **f-j**, *tko^{25t};sss^{p1}* flies exhibit prolonged seizures as compared to mutant *tko^{25t}* flies. n = 18 vials/condition. n = 33-44 flies/condition. Two-group two-tailed t-test, one-way ANOVA with Dunnett's or Tukey's multiple comparisons test, or Kruskal-Wallis with Dunn's multiple comparisons (for seizure percentage) test was used. **p<0.01, ***p<0.001, ****p<0.0001**.** Data are presented as mean values ± SEM.

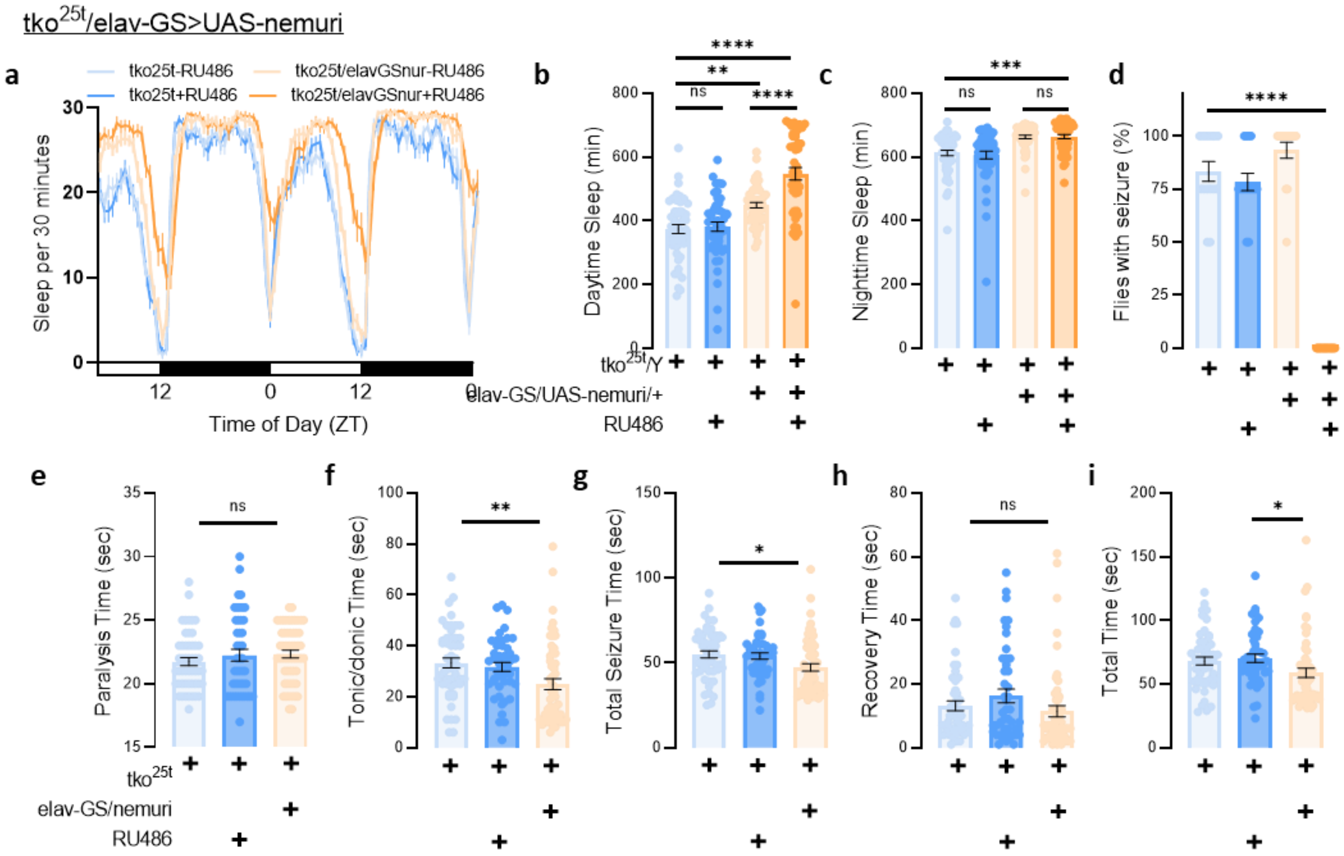

Supplementary Fig. 3 │Sleep induction through *nemuri* overexpression leads to decreased seizure likelihood and severity. a-c, In *tko*^{25t} mutant flies, overexpression of UAS-*nemuri* using an inducible, pan-neuronal Gal4 driver (*elav*-GeneSwitch, *elav*-GS) by feeding flies the GeneSwitch activator RU486 leads to increased daytime and nighttime sleep n = 45-48 flies/condition. **d-i**, *nemuri* overexpression leads complete suppression of seizures. Even in the absence of the GeneSwitch activator RU486, there are decreased seizure times, likely due to leaky nemuri expression. n = 15 vials/condition. n = 45-55 flies/condition. One-way ANOVA with Dunnett's multiple comparisons test or Kruskal-Wallis with Dunn's multiple comparisons test (for seizure percentage) was used. *p<0.05, **p<0.01, ***p<0.001, ****p<0.0001. Data are presented as mean values ± SEM.

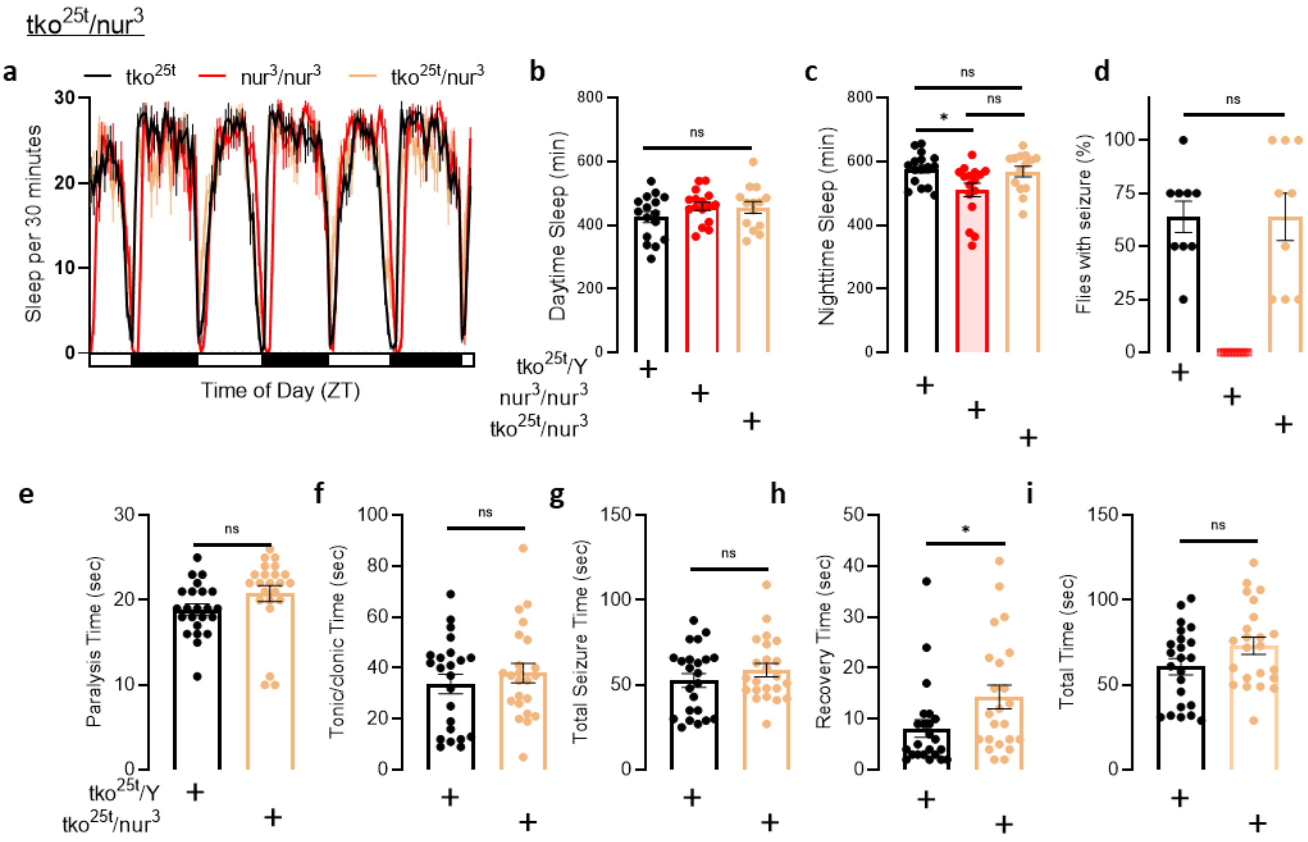

Supplementary Fig. 4 │ Crossing *tko^{25t}* mutant flies with *nemuri* mutant flies has little effects on sleep or seizure severity. a-c, Crossing *tko^{25t}* mutant flies with *nemuri* mutant flies (*nur^{3}*) was not associated with significant changes in sleep duration. n = 14-16 flies. **d-i**, There was no significant evidence of a change in seizure likelihood and duration among *tko^{25t};nur^{3}* mutant flies. n = 9 vials/condition. n = 23 flies/condition. Two-group two-tailed t-test, one-way ANOVA with Dunnett's multiple comparisons test, or Kruskal-Wallis with Dunn's multiple comparisons test (for seizure percentage) was used. *p<0.05. Data are presented as mean values ± SEM.

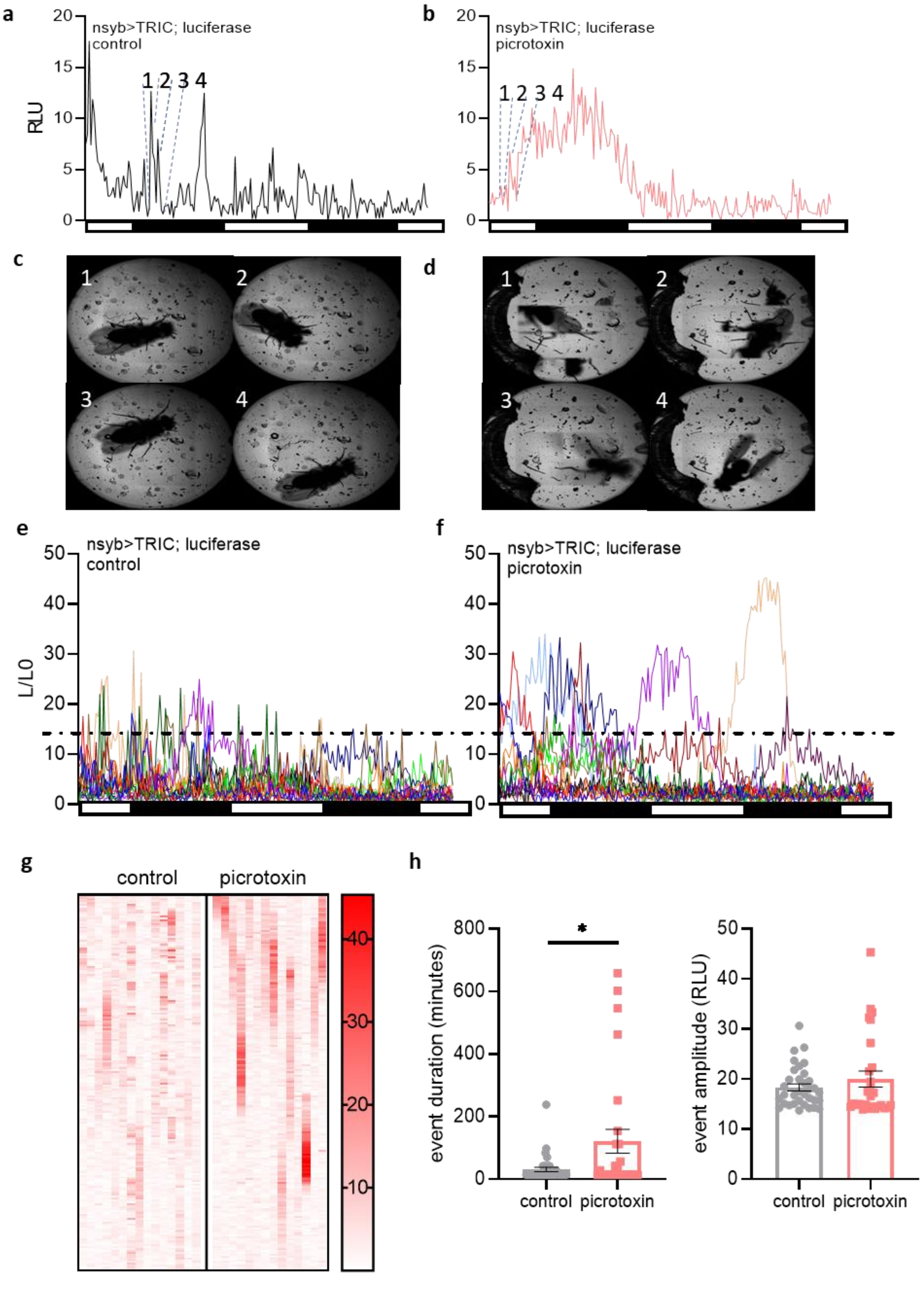

Supplementary Fig. 5 │ Spontaneous tonic-clonic seizures in *Drosophila* are associated with prolonged, hypersynchronous neuronal activity. nsyb-Gal4>UAS-TRIC-luciferase flies were treated with vehicle or picrotoxin and monitored for 48 hours with imaging of fly posture and

detection of luminescence. **a**, **c**, Representative control nsyb-Gal4>UAS-TRIC-luciferase fly treated with vehicle demonstrates that transient spikes in neuronal activity does not correlate with hyperkinetic movements. Timepoints *1, 2, 3,* and *4* in **a** demonstrate a sample spike in luminescence does not correlate with tonic-clonic movements in fly at the same timepoints in **c**. **b, d**, Representative nsyb-Gal4>UAS-TRIC-luciferase fly treated with picrotoxin demonstrates prolonged increases in neuronal activity correlates with hyperkinetic movements. Timepoints *1, 2, 3,* and *4* in **b** demonstrate a prolonged increase in luminescence that correlates with tonic-clonic movements at the same timepoints in **d**. Note that each well containing a fly is constructed from a composite of multiple images of the well. When flies have quick convulsive movements, wings and legs appear blurred, and the fly body appears fragmented. **e-f**, Each colored line depicts an individual fly monitored over two days from a representative experiment. Picrotoxin-treated nsyb-Gal4>UAS-TRIC-luciferase flies exhibit prolonged increases in neuronal activity as measured by luminescence recordings. Recordings are normalized to baseline and presented as L/L_0. **g**, Heatmap of same flies as in E-F, but each column demonstrates luminescence over time. Flies treated with picrotoxin exhibit prolonged hypersynchronous neuronal activity. **h**, Quantification of spikes in luminescence demonstrate that picrotoxin induces prolonged bouts of neuronal activity. n = 14-15 flies/condition. Two-group two-tailed t-test was used. *$p<0.05$. Data are presented as mean values ± SEM.

Anti-Seizure Medication Treatment

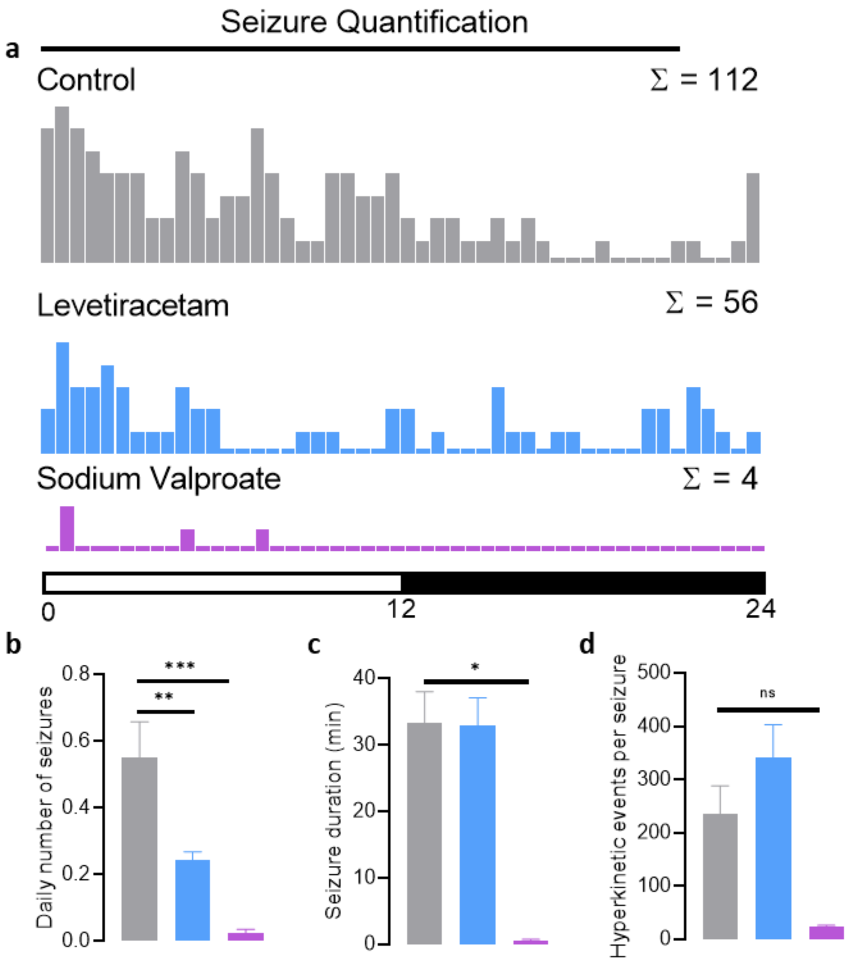

Supplementary Fig. 6 │ Spontaneous seizures are ameliorated with conventional anti-seizure medications. **a**, Wild-type Canton-S flies were video recorded in a 48-well plate containing picrotoxin. Spontaneous seizures were counted across 48 30-minute time bins for each treatment condition - control, levetiracetam (*blue*), and sodium valproate (*purple*). The anti-seizure medications levetiracetam and sodium valproate decreased the number of spontaneous seizures (Σ). n=48 flies/condition. **b**, Average number of seizures per day per fly is decreased in levetiracetam (*blue*) and sodium valproate (*purple*) groups as compared to control. n=48 flies/condition. **c**, Average seizure duration is decreased with sodium valproate treatment (*purple*) as compared to control. n=4-87 seizures/condition. **d**, Average number of hyperkinetic events per seizures is decreased with sodium valproate treatment (*purple*) as compared to control. n=4-87 seizures/condition. Negative binomial model with Wald test for daily number of seizures or mixed effects model (for seizure durations and hyperkinetic events) was used. *$p<0.05$, **$p<0.01$, ***$p<0.001$. Data are presented as mean values ± SEM.

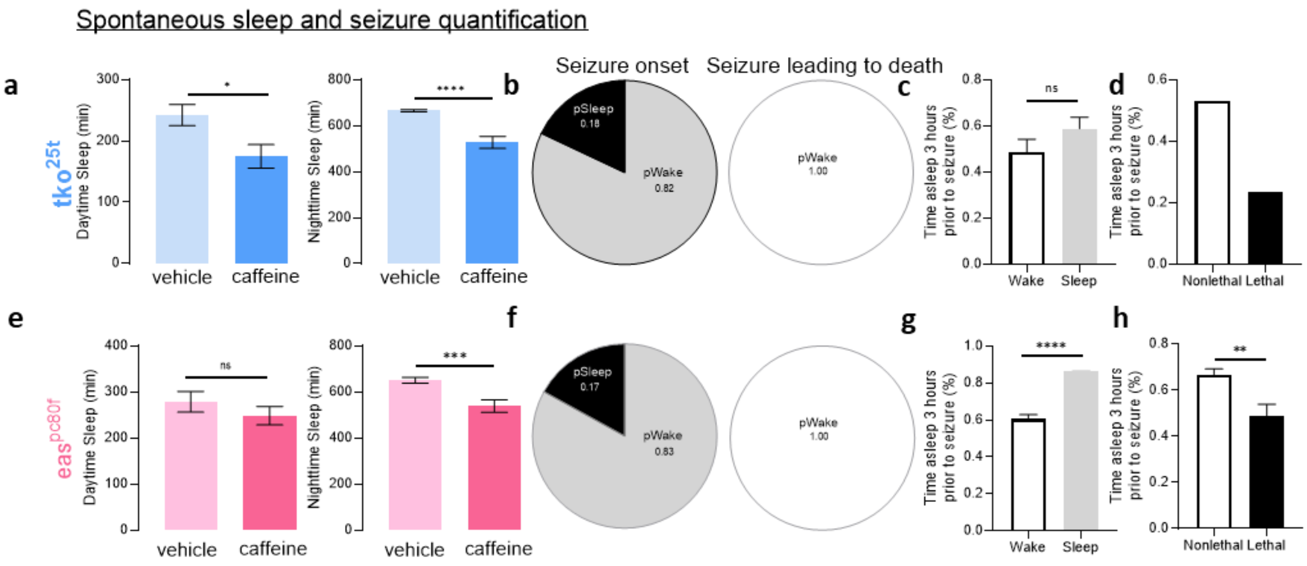

Supplementary Fig. 7 │ A protocol for detection of spontaneous seizures in *Drosophila* reveals spontaneous seizures after sleep restriction. a, *tko*^{25t} mutant flies treated with caffeine exhibit decreased mean levels of daytime and nighttime sleep as assessed with video tracking. (Lighter colors are with vehicle and darker colors are with caffeine treatment). n = 29-32 flies/condition. **b**, Probability of the fly being in a sleep or wake state at the time of seizure onset for all seizures (left) and seizures that end in death (right). *tko*^{25t} mutant flies more frequently exhibit seizures during wakefulness. Lethal seizures always occur during wakefulness. **c**, Sleep history is similar between *tko*^{25t} mutant flies that have seizures during wakefulness versus those that seize during sleep. n = 2-9 seizures/condition. **d**, Lethal seizures correlate with less time asleep preceding seizures. Error bars not depicted given only a single seizure in *tko*^{25t} mutant flies led to death. n = 1-10 seizures/condition. **e**, *eas*^{pc80f} mutant flies treated with caffeine exhibit decreased nighttime sleep as assessed with video tracking. n = 29-32 flies/condition. **f**, *eas*^{pc80f} mutant flies more frequently exhibit seizures during wakefulness. Lethal seizures always occur during wakefulness. **g**, Seizures occurring in sleep correlate with a greater percentage of the preceding 3 hours spent in sleep. n = 14-69 seizures/condition. **h**, Lethal seizures correlate with less time asleep preceding seizures in *eas*^{pc80f} flies. n = 9-74 seizures/condition. Two-group two-tailed t-test was used. $*p<0.05$, $**p<0.01$, $***p<0.001$, $****p<0.0001$. Data are presented as mean values ± SEM.

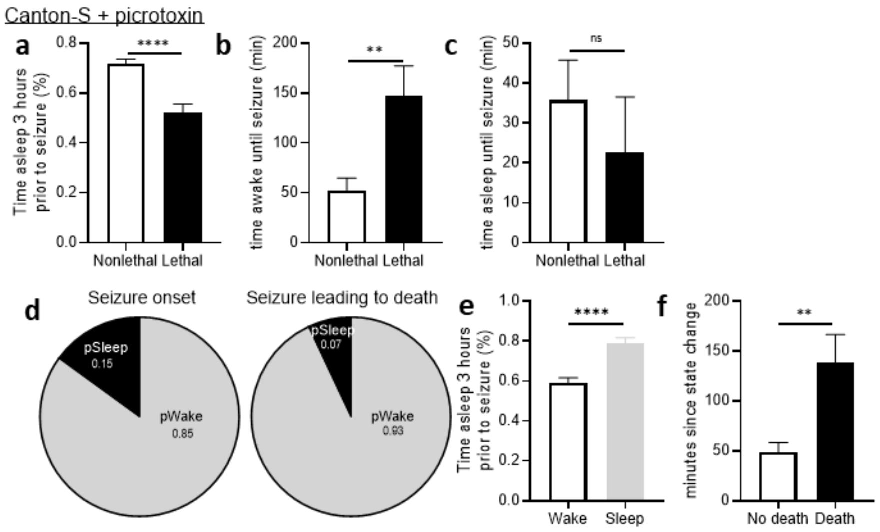

Supplementary Fig. 8 │A protocol for detection of spontaneous seizures in *Drosophila* reveals spontaneous seizures after sleep restriction. a, Retrospective analysis of wild-type Canton-S flies treated with picrotoxin demonstrates that lethal seizures occur after decreased sleep in the 3 preceding hours as compared to nonlethal seizures. n=41-44 seizures/condition. **b**, On average, wild-type Canton-S flies treated with picrotoxin with lethal seizures had longer wake times prior to seizure onset. n=34-38 seizure/condition. **c**, The duration of sleeping in wild-type Canton-S flies asleep at seizure onset did not predict seizure lethality. n=3-10 seizures/condition. **d**, Wild-type Canton-S flies treated with picrotoxin were more likely to have seizures during wakefulness, and lethal seizures were more likely to occur during wakefulness. **e**, Seizures occurring in sleep correlate with a greater percentage of the preceding 3 hours spent in sleep. n = 13-72 seizures/condition. **f**, Lethal seizures are more likely to occur as duration since a wake-to-sleep or sleep-to-wake transition increases. n = 41-44 seizures/condition. Two-group two-tailed t-test was used. $^{**}p<0.01$, $^{****}p<0.0001$. Data are presented as mean values ± SEM.

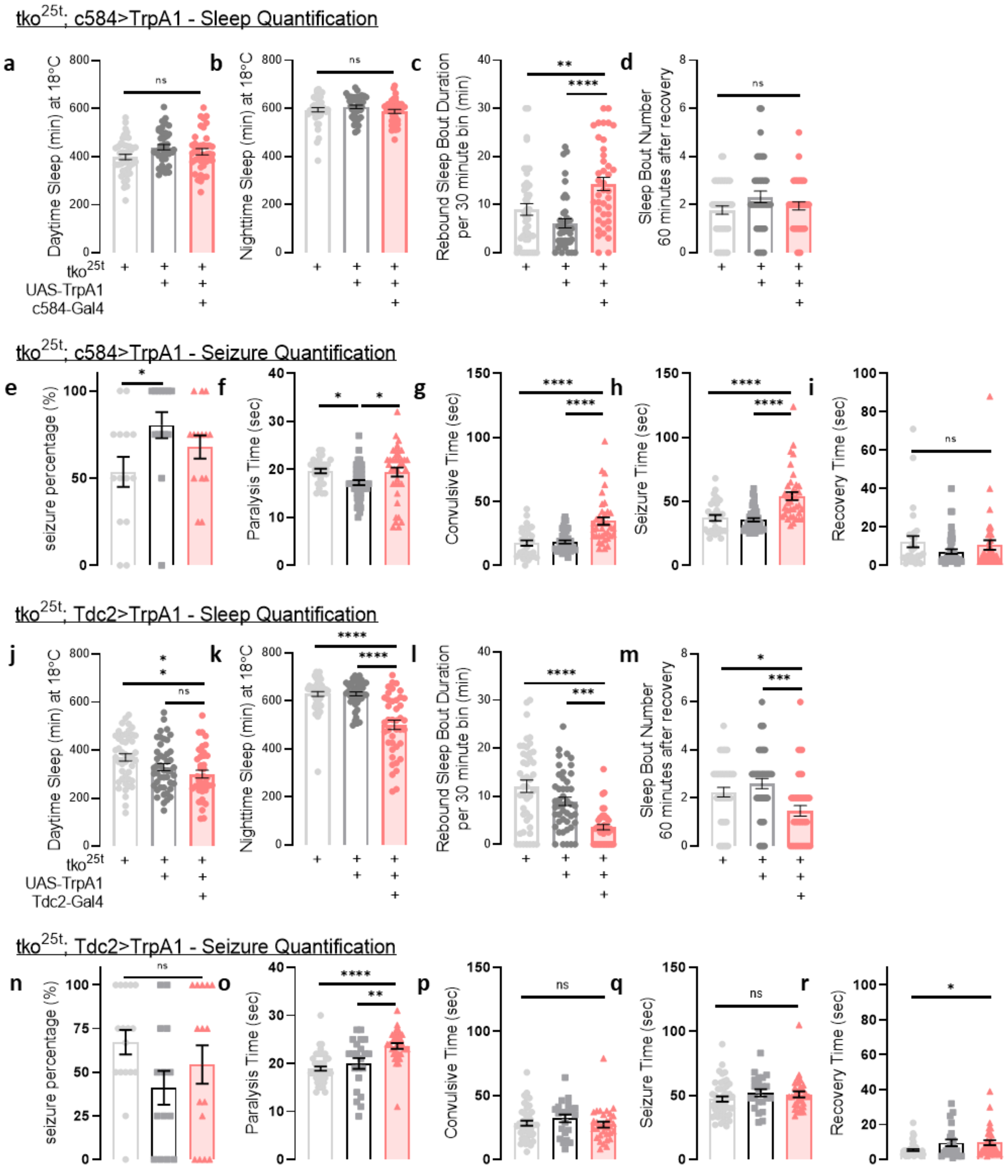

Supplementary Fig. 9 │ Sleep restriction using manipulations that increase sleep rebound leads to more severe induced seizures in *tko^{25t}* mutant flies. a, Before shifting to 30°C, there is no difference in daytime sleep in *tko^{25t}*; c584-Gal4>UAS-TrpA1 flies. **b**, After shifting back from 30°C, there is no difference in nighttime sleep. **c**, After returning to 18°C, there is a prolongation of sleep bout duration in the ensuing 60 minutes, indicative of increased sleep consolidation. n =

40-43 flies/condition. **d**, After returning to 18°C, there is no change in the total number of sleep bouts in the ensuing 60 minutes. n = 40-43 flies/condition. **e-i**, tko^{25t}; c584-Gal4>UAS-TrpA1 flies exhibit prolonged seizures after TrpA1 activation at 30°C for 24 hours. n = 14 vials/condition. n = 30-45 flies/condition. **j**, Before shifting to 30°C, daytime sleep in tko^{25t}; Tdc2-Gal4>UAS-TrpA1 flies is comparable to genetic controls. **k**, After shifting back from 30°C, there continues to be a decrease in nighttime sleep in tko^{25t}; Tdc2-Gal4>UAS-TrpA1 flies. **l**, After returning to 18°C, there is a decrease in sleep bout duration in the ensuing 60 minutes. n=41-46 flies/condition. **m**, After returning to 18°C, there is a decrease in the total number of sleep bouts in the ensuing 60 minutes. n=41-46 flies/condition. **n-r**, tko^{25t}; Tdc2-Gal4>UAS-TrpA1 flies exhibit no change in convulsive (tonic/clonic) times and seizure times after TrpA1 activation at 30°C for 24 hours but show prolonged paralysis and recovery times. n = 14-17 vials/condition. n = 22-45 flies/condition. One-way ANOVA with Dunnett's or Tukey's multiple comparisons test or Kruskal-Wallis with Dunn's multiple comparisons (for seizure percentage) test was used. *p<0.05, **p<0.01, ***p<0.001, ****p<0.0001. Data are presented as mean values ± SEM.

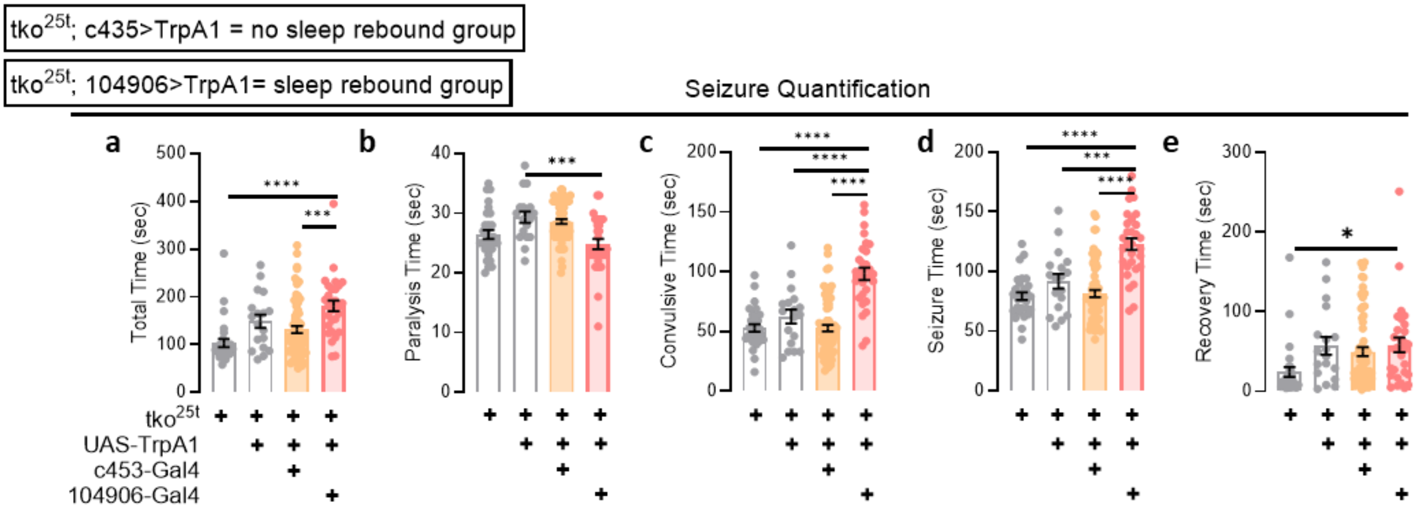

Supplementary Fig. 10 │ Sleep restriction using genetic drivers associated with sleep rebound lead to more severe induced seizures in *tko^{25t}* mutant flies. a-e, *tko^{25t}*; 104906-Gal4>UAS-TrpA1 flies, which are associated with sleep loss followed by sleep rebound, exhibit prolonged convulsive (tonic/clonic) times and seizure times after TrpA1 activation at 30°C for 24 hours as compared to *tko^{25t}*; c453-Gal4>UAS-TrpA1 flies, which are associated with sleep loss without sleep rebound. n = 18-67 flies/condition. One-way ANOVA with Dunnett's or Tukey's multiple comparisons test was used. *p<0.05, ***p<0.001, ****p<0.0001. Data are presented as mean values ± SEM.

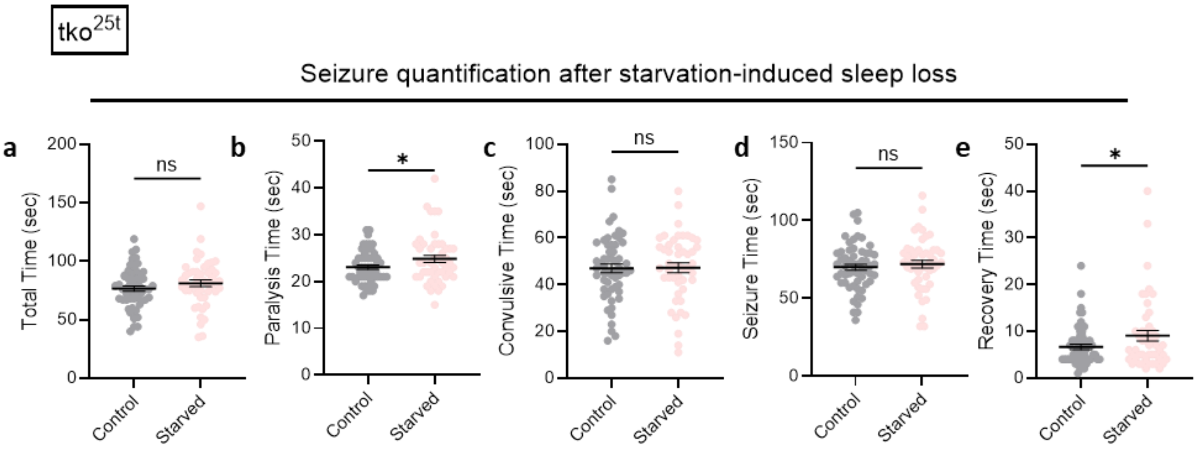

Supplementary Fig. 11 │ Sleep restriction after starvation does not increase convulsive time or induced seizure time in *tko*^{25t} mutant flies. a-e, *tko*^{25t} flies were starved for 12 hours then seizures were induced. There was no significant prolongation of convulsive (tonic/clonic) or total seizure time as compared to fed flies. Starvation is associated with sleep loss without sleep rebound[33]. n = 48-56 flies/condition. Unpaired two-tailed t-test was used. *p<0.05. Data are presented as mean values ± SEM.

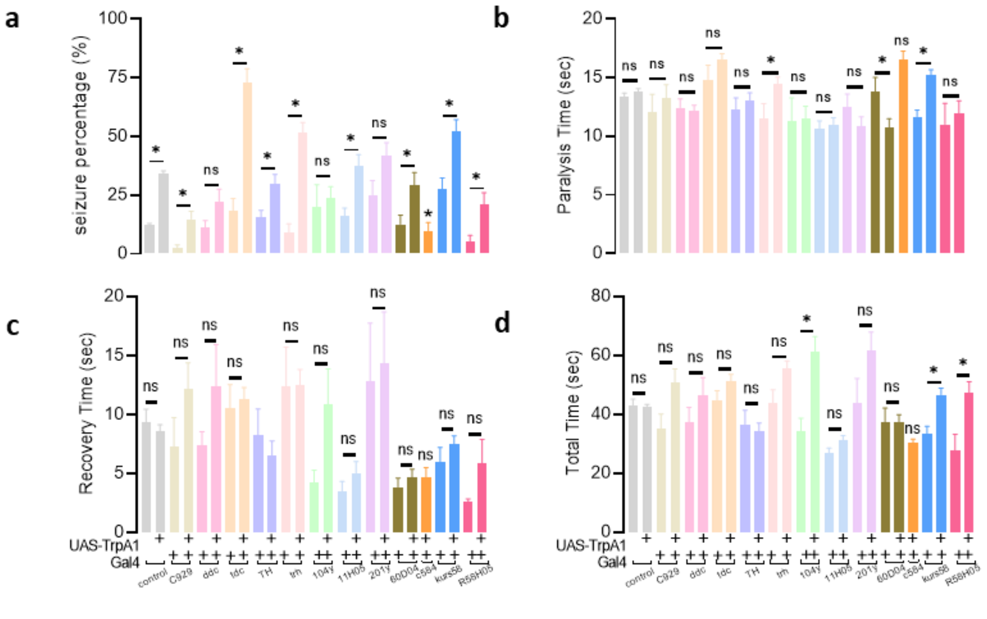

Supplementary Fig. 12 │ A Gal4 screen of wake- and sleep-promoting circuits and cellular subpopulations reveals that acute activation of sleep-promoting circuits worsens seizures. To drive TrpA1, Gal4 drivers were selected for peptidergic neurons (C929-Gal4), serotonergic/dopaminergic neurons (Ddc-Gal4), octopaminergic neurons (Tdc2-Gal4), dopaminergic neurons (TH-Gal4), serotonergic neurons (trh-Gal4), dorsal fan-shaped body (104y-Gal4), wake-promoting neurons (11H05-Gal4, 60D04-Gal4, c584-Gal4), mushroom body (201y-Gal4), pars intercerebralis (kurs58-Gal4), and ellipsoid body (R58H05-Gal4). **a**, Percentage of flies exhibiting seizures after mechanical stimulus. **b**, Paralysis time after seizure induction **c**, Recovery time after seizure induction. **d**, Total time until recovery after seizure induction. n = 4-77. Values shown are means and standard errors of the mean. Mann-Whitney test (for seizure percentages) or two-group two-tailed t-test was used. *p<0.05. Data are presented as mean values ± SEM.

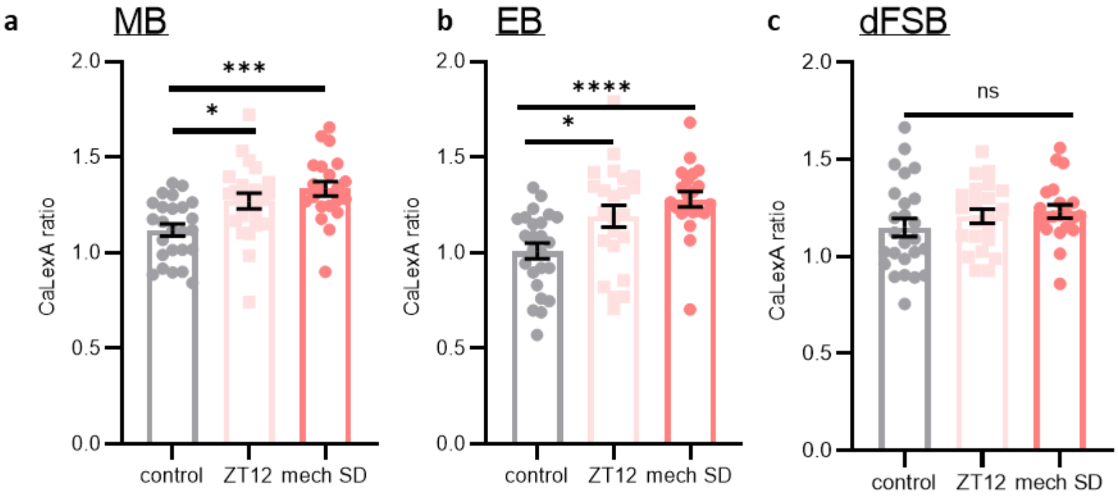

Supplementary Fig. 13 | High sleep drive correlates with increased activity of sleep-promoting brain regions. a-c, At the end of the day (ZT12) and after a night of mechanical sleep deprivation in nsyb-Gal4>CaLexA flies, there is increased CaLexA (GFP:RFP) signal in the mushroom body (MB) and ellipsoid body (EB) but not in the dorsal fan-shaped body (dFSB). n = 21-25 brains/condition. One-way ANOVA with Tukey's multiple comparisons test was used. **p<0.01, ****p<0.0001. Data are presented as mean values ± SEM.

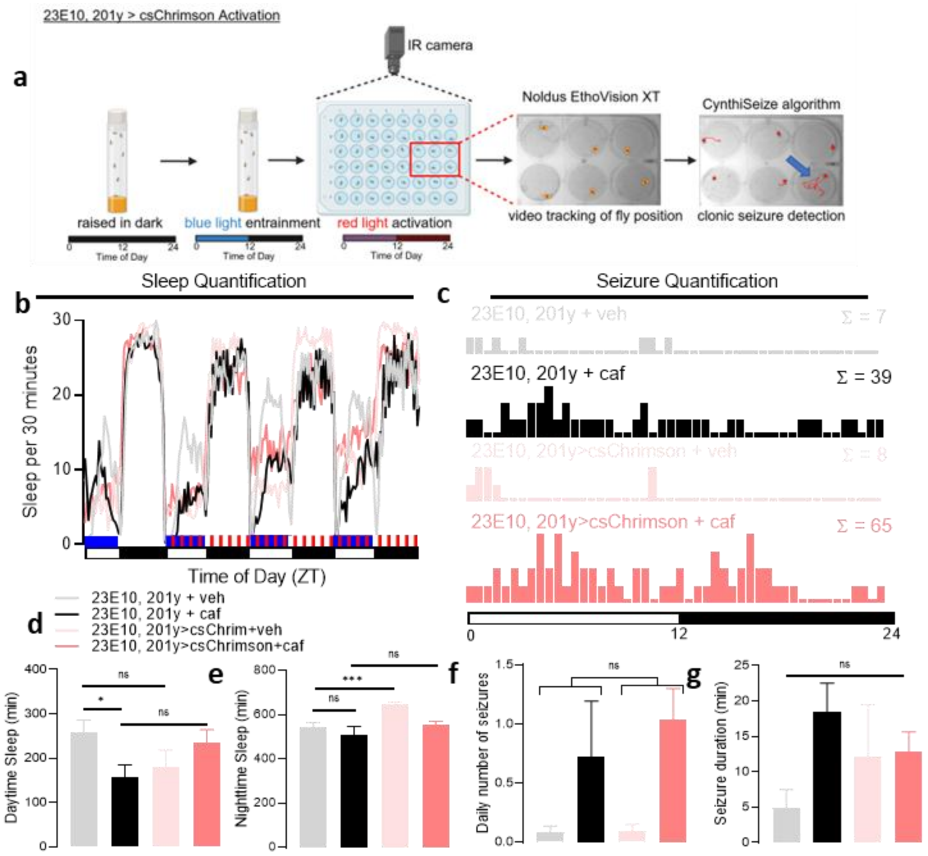

Supplementary Fig. 14 │Optogenetic modulation of sleep-promoting circuits controls seizure risk in the setting of sleep loss. a, Experimental protocol showing flies were raised in darkness, entrained in blue light, then placed into 24- or 48-well plates for chronic video monitoring with red light stimulation. Fly positions over time were converted into XY coordinates, and a "CynthiSeize" algorithm was developed to identify seizures. Created in BioRender. Sehgal, A. (2025) https://BioRender.com/uir8dyn **b**, **d**, **e**, Sleep quantification after 23E10-Gal4, 201y-Gal4>csChrimson activation. Caffeine decreases mean daytime sleep duration, and 23E10-Gal4, 201y-Gal4>csChrimson increases mean nighttime sleep duration. n=17-18 flies/condition. **c**, **f**, **g**, Seizure quantification after 23E10-Gal4, 201y-Gal4>csChrimson activation. Caffeine increases seizure frequency and is not additive with 23E10-Gal4, 201y-Gal4>csChrimson activation. n=17-18 flies/condition. One-way ANOVA with Dunnett's T3 multiple comparisons adjustment, Kruskal-Wallis test with Dunn's multiple comparisons adjustment, negative binomial model with Wald test (for daily number of seizures), or mixed effects model (for seizure durations) was used. *p<0.05, ***p<0.001. Data are presented as mean values ± SEM.

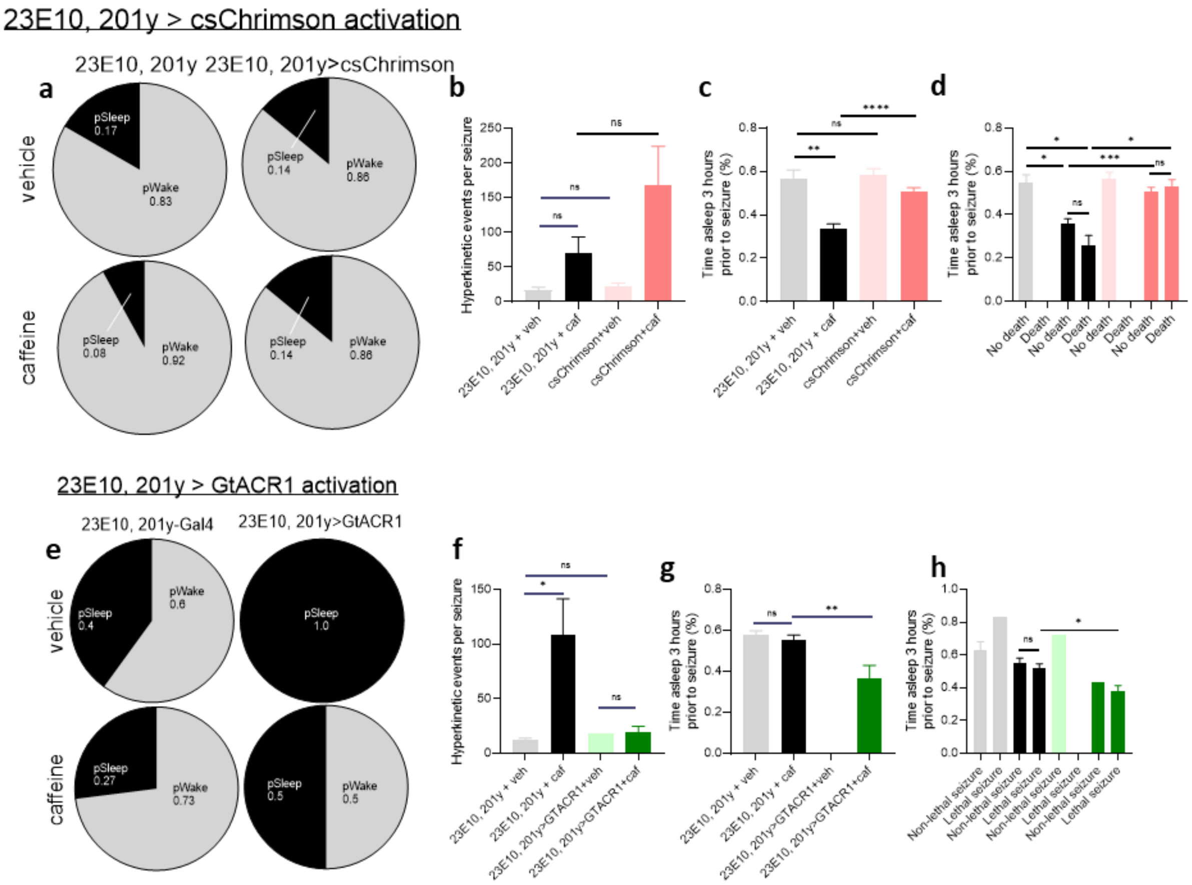

Supplementary Fig. 15 │ Optogenetic modulation of sleep-promoting circuits changes seizure risk. a, Likelihood of being awake (pWake) or asleep (pSleep) at seizure onset. Seizures are more likely to occur during wakefulness even after csChrimson activation, with or without caffeine treatment. **b**, There is no significant evidence that the number of hyperkinetic events per seizure change after caffeine treatment or after csChrimson activation. n = 6-49 seizures/condition. **c**, Seizures occurring with caffeine treatment during wakefulness are more likely to occur after decreased sleep in the 3 hours preceding seizures. Compared to 23E10-Gal4, 201y-Gal4 genetic control flies treated with caffeine, 23E10-Gal4, 201y-Gal4>csChrimson activation with caffeine treatment increases sleep quantity prior to seizure onset. n = 5-42 seizures/condition **d**, In flies treated with caffeine, lethal and non-lethal seizures are more likely to occur after decreased sleep in the preceding 3 hours. csChrimson activation increases sleep prior to seizure onset. n = 0-36 seizures/condition. **e**, Likelihood of being awake (pWake) or asleep (pSleep) at seizure onset. After 23E10-Gal4, 201y-Gal4>GtACR1 activation, a high proportion of seizures occur during sleep than without GtACR1 activation. **f**, Seizures occurring with caffeine treatment exhibit an increased number of hyperkinetic events per seizure n = 1-15 seizures/condition. **g**, In awake flies, GtACR1 activation with caffeine treatment decreases the sleep amount prior to seizure onset. n = 0-11 seizures/condition. **h**, Among flies treated with caffeine, flies with lethal seizures sleep less with GtACR1 activation. n = 0-8 seizures/condition. One-way ANOVA with Dunnett's T3 multiple comparisons test or mixed effects model (for hyperkinetic events per seizure) was used. *p<0.05, **p<0.01, ***p<0.001, ****p<0.0001. Data are presented as mean values ± SEM.

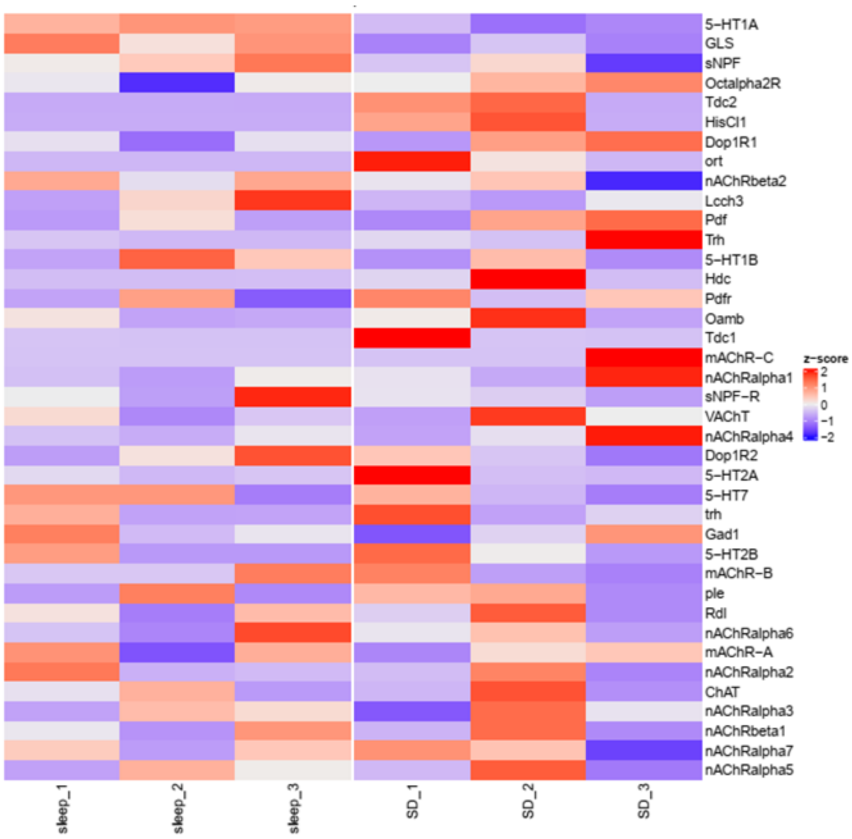

Supplementary Fig. 16 │Heatmap of all samples demonstrating 5HT1A is downregulated after sleep loss. Transcriptomic analysis of the dorsal fan-shaped body after sleep-restriction reveals downregulation of 5HT1A, but not other neurotransmitter receptors or rate-limiting enzymes. "*sleep_1*", "*sleep_2*", and "*sleep_3*" indicate control flies allowed to sleep. "*SD_1*", "*SD_2*", and "*SD_3*" indicate experimental flies that were mechanically sleep-deprived. n=3 samples/condition. Significance testing with two-tailed t-test. *p<0.05.

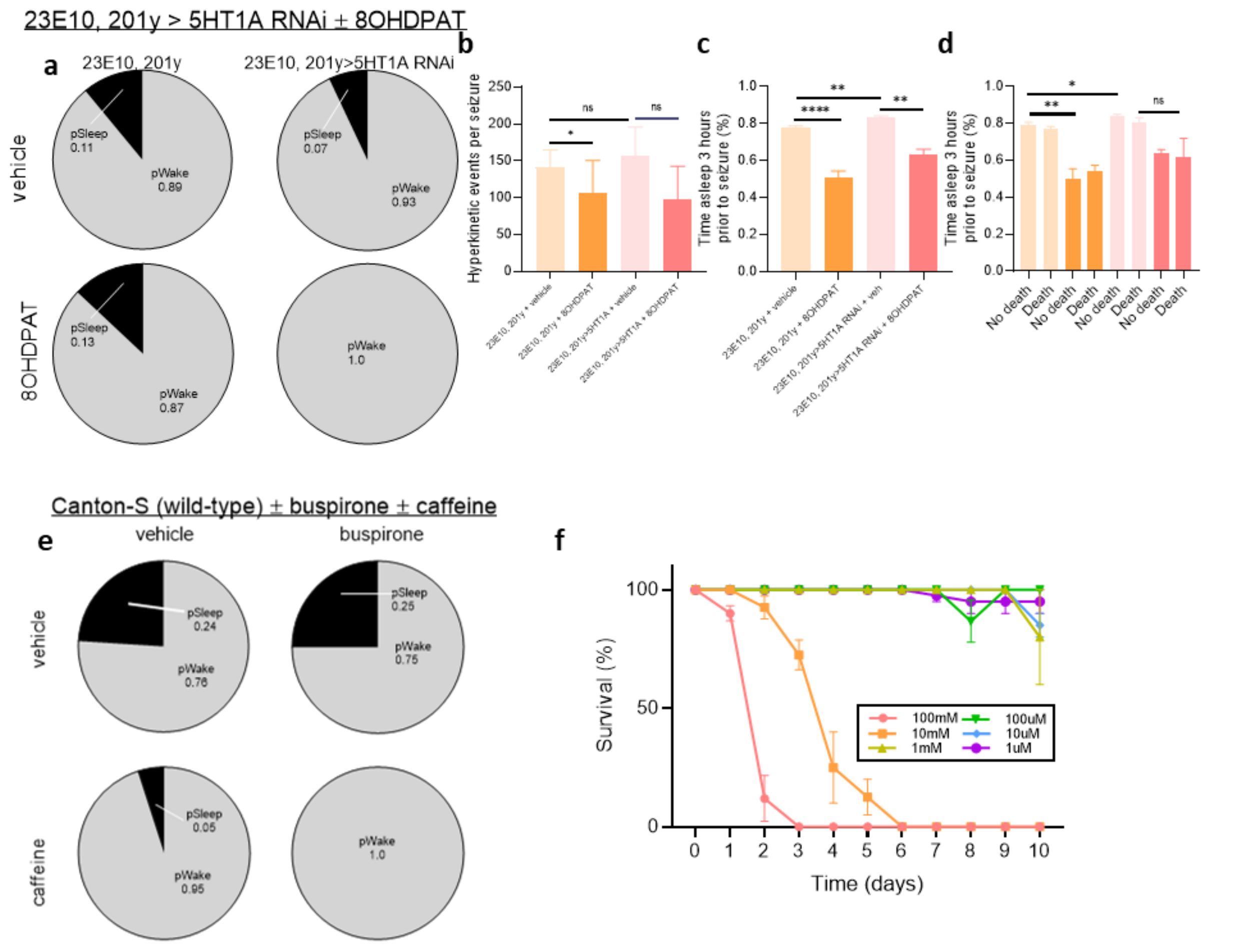

Supplementary Fig. 17 │ Serotonergic modulation of sleep-promoting circuits changes seizure risk. a, Likelihood of being awake (pWake) or asleep (pSleep) at seizure onset. Seizures are more likely to occur during wakefulness even after 8-OH-DPAT, a selective 5HT1A receptor agonist, treatment or 5HT1A RNAi-mediated knockdown. **b**, There is no significant evidence of a change in the number of hyperkinetic events per seizure after 8-OH-DPAT treatment or 5HT1A RNAi-mediated knockdown. n = 6-76 seizures/condition. **c**, 8-OH-DPAT treatment decreases the duration of sleep in awake flies prior to seizure onset. n = 6-68 seizures/condition. **d**, 8-OH-DPAT treatment decreases the duration of sleep prior to onset of lethal and non-lethal seizures. n = 2-46 seizures/condition. **e**, Likelihood of being awake (pWake) or asleep (pSleep) at seizure onset. Seizures are more likely to occur during wakefulness even after buspirone treatment. **f**, Wild-type Canton-S flies were treated with 1 uM, 10 uM, 100 uM, 1 mM, 10 mM, and 100 mM buspirone for 10 days and the number of surviving flies were counted. n = 4 vials/condition with 40 flies total/condition. One-way ANOVA with Dunnett’s T3 multiple comparisons test or mixed effects model (for hyperkinetic events per seizure) was used. *$p<0.05$, **$p<0.01$, ***$p<0.001$, ****$p<0.0001$. Data are presented as mean values ± SEM.

Supplementary References

1. Shi, M., Yue, Z., Kuryatov, A., Lindstrom, J. M. & Sehgal, A. Identification of Redeye, a new sleep-regulating protein whose expression is modulated by sleep amount. *Elife* **3**, e01473 (2014).
2. Royden, C. S., Pirrotta, V. & Jan, L. Y. The tko locus, site of a behavioral mutation in D. melanogaster, codes for a protein homologous to prokaryotic ribosomal protein S12. *Cell* **51**, 165–173 (1987).
3. Koh, K. *et al*. Identification of SLEEPLESS, a sleep-promoting factor. *Science* **321**, 372–376 (2008).
4. Hamada, F. N. *et al*. An internal thermal sensor controlling temperature preference in Drosophila. *Nature* **454**, 217–220 (2008).
5. Schwarz, J. E., King, A. N., Hsu, C. T., Barber, A. F. & Sehgal, A. Hugin (+) neurons provide a link between sleep homeostat and circadian clock neurons. *Proc. Natl. Acad. Sci. U. S. A.* **118**, 10.1073/pnas.2111183118 (2021).
6. Masuyama, K., Zhang, Y., Rao, Y. & Wang, J. W. Mapping neural circuits with activity-dependent nuclear import of a transcription factor. *J. Neurogenet.* **26**, 89–102 (2012).
7. Toda, H., Williams, J. A., Gulledge, M. & Sehgal, A. A sleep-inducing gene, nemuri, links sleep and immune function in Drosophila. *Science* **363**, 509–515 (2019).
8. Nall, A. H. *et al*. Caffeine promotes wakefulness via dopamine signaling in Drosophila. *Sci. Rep.* **6**, 20938 (2016).
9. Thimgan, M. S., Suzuki, Y., Seugnet, L., Gottschalk, L. & Shaw, P. J. The perilipin homologue, lipid storage droplet 2, regulates sleep homeostasis and prevents learning impairments following sleep loss. *PLoS Biol.* **8**, e1000466. doi: 10.1371/journal.pbio.1000466 (2010).
10. Shaw, P. J., Cirelli, C., Greenspan, R. J. & Tononi, G. Correlates of sleep and waking in Drosophila melanogaster. *Science* **287**, 1834–1837 (2000).
11. Hendricks, J. C. *et al*. Rest in Drosophila is a sleep-like state. *Neuron* **25**, 129–138 (2000).
12. Lenz, O., Xiong, J., Nelson, M. D., Raizen, D. M. & Williams, J. A. FMRFamide signaling promotes stress-induced sleep in Drosophila. *Brain Behav. Immun.* **47**, 141–148 (2015).
13. Singh, P., Mohammad, F. & Sharma, A. Transcriptomic analysis in a Drosophila model identifies previously implicated and novel pathways in the therapeutic mechanism in neuropsychiatric disorders. *Front. Neurosci.* **5**, 161 (2011).
14. Yi, J. *et al*. Sodium valproate alleviates neurodegeneration in SCA3/MJD via suppressing apoptosis and rescuing the hypoacetylation levels of histone H3 and H4. *PLoS One* **8**, e54792 (2013).
15. Dokucu, M. E., Yu, L. & Taghert, P. H. Lithium- and valproate-induced alterations in circadian locomotor behavior in Drosophila. *Neuropsychopharmacology* **30**, 2216–2224 (2005).
16. Johnson, O., Becnel, J. & Nichols, C. D. Serotonin 5-HT(2) and 5-HT(1A)-like receptors differentially modulate aggressive behaviors in Drosophila melanogaster. *Neuroscience* **158**, 1292–1300 (2009).
17. Polter, A. M. & Li, X. 5-HT1A receptor-regulated signal transduction pathways in brain. *Cell. Signal.* **22**, 1406–1412 (2010).
18. Garbe, D. S. *et al*. Context-specific comparison of sleep acquisition systems in Drosophila. *Biol. Open* **4**, 1558–1568 (2015).
19. Mohammad, F. *et al*. Optogenetic inhibition of behavior with anion channelrhodopsins. *Nat. Methods* **14**, 271–274 (2017).

20. Klaassen, A. *et al*. Seizures and enhanced cortical GABAergic inhibition in two mouse models of human autosomal dominant nocturnal frontal lobe epilepsy. *Proc. Natl. Acad. Sci. U. S. A.* **103**, 19152–19157 (2006).
21. Steinlein, O. K. *et al*. A missense mutation in the neuronal nicotinic acetylcholine receptor alpha 4 subunit is associated with autosomal dominant nocturnal frontal lobe epilepsy. *Nat. Genet.* **11**, 201–203 (1995).
22. Yap, M. H. W. *et al*. Oscillatory brain activity in spontaneous and induced sleep stages in flies. *Nat. Commun.* **8**, 1815–y (2017).
23. Vyazovskiy, V. V., Kopp, C., Bosch, G. & Tobler, I. The GABAA receptor agonist THIP alters the EEG in waking and sleep of mice. *Neuropharmacology* **48**, 617–626 (2005).
