# Supplementary material for: Sleep drive, not total sleep amount, increases seizure risk
Source: Nat Commun. 2025 Jul 29;16:6967. doi: 10.1038/s41467-025-62311-x (PMC12307685; doi:10.1038/s41467-025-62311-x)
Supplement: Supplementary file 2 — Description of Additional Supplementary Files [file 41467_2025_62311_MOESM2_ESM.pdf]

Description of Additional Supplementary Files

File name: Supplementary Movie 1
Description: Sample video depicting induced bang-sensitive seizures in *tko*^{25t} mutant flies. Four *tko*^{25t} flies were placed into each vial. First vial contains vehicle, second vial contains caffeine, and third vial contains gaboxadol. Flies were exposed to a mechanical stimulus on a vortexer prior to start of video recording. Seizures were quantified for atonic ("paralysis"), tonic/clonic ("convulsive"), and recovery ("postictal") phases. Caffeine-treated *tko*^{25t} flies (second vial) demonstrated prolonged seizure times.

File name: Supplementary Movie 2
Description: Sample video depicting a spontaneous seizure in wild-type Canton-S fly treated with picrotoxin. Canton-S flies were loaded into each well of a 48-well plate. Control flies are in the first two rows (*rows A and B*). Flies treated with levetiracetam, an anti-seizure medication, are in the middle two rows (*rows C and D*). Flies treated with sodium valproate, an anti-seizure medication, are in the bottom two rows (*rows E and F*). Well #A4 (*identified with red circle)* demonstrates a spontaneous tonic-clonic seizure.

File name: Supplementary Movie 3
Description: Sample video depicting a spontaneous seizure in wild-type Canton-S fly treated with picrotoxin with position tracking visualization. Same video as *Supplementary Movie 2* except fly position over the previous 10 seconds is indicated with red line. As indicated by the fly exhibiting a tonic-clonic seizure in well #A4 (*identified with red circle*), movement characteristics are distinct from baseline fly movements.

File name: Supplementary Movie 4
Description: Sample video depicting a spontaneous seizure in wild-type Canton-S fly treated with picrotoxin recorded using visible light. Canton-S flies were loaded into each well of a 48-well plate and fed picrotoxin. Instead of video recording with an IR filter as seen in Supplementary Movie 2 and Supplementary Movie 3, flies were recorded using visible light for visualization purposes. This video was taken after the conclusion of typical experimental recording period and observed by the experimenter without using video tracking software. Well #B5 (*identified with red circle)* demonstrates a spontaneous tonic-clonic seizure. Lethality was noted after picrotoxin-induced seizures and found to be associated with sleep (Supplementary Fig. 8).
